# 170. Antimicrobial Use Before and During COVID-19 – Data from 108 VA Facilities

**DOI:** 10.1093/ofid/ofab466.372

**Published:** 2021-12-04

**Authors:** Matthew B Goetz, Matthew B Goetz, Tina M Willson, Vanessa W Stevens, Christopher J Graber, Michael Rubin

**Affiliations:** 1 VA Greater Los Angeles Healthcare System and David Geffen School of Medicine at UCLA, VA-CDC Practice-Based Research Network, Los Angeles, California; 2 University of Utah, Salt Lake City, Utah; 3 VA Salt Lake City Health Care System, Salt Lake City, Utah; 4 VA Greater Los Angeles Healthcare System/UCLA, Los Angeles, California; 5 VA Salt Lake City HCS, Salt Lake City, UT

## Abstract

**Background:**

Increased antibiotic prescribing rates during the early phases of the COVID-19 pandemic have been widely reported. We previously reported that while both antibiotic days of therapy (DOT) and total days present (DP) declined in the first 5 months of 2020 at Veterans Affairs (VA) acute care facilities nationwide relative to the comparable period in 2019, antibiotic DOT per 1000 DP increased by 11.3%, largely reversing declines in VA antimicrobial utilization from 2015 – 2019. We now evaluate whether these changes in antibiotic use persisted throughout the COVID-19 pandemic.

**Methods:**

Data on antibacterial use, patient days present, and COVID-19 care for acute inpatient care units in 108 VA level 1 and 2 facilities were extracted through the VA Informatics and Computing Infrastructure; level 3 facilities which provide limited acute inpatient services were excluded. DOT per 1000 DP were calculated and stratified by CDC-defined antibiotic classes.

**Results:**

From 1/2020 to 2/2021, care for 34,096 COVID-19 patients accounted for 13% of all acute inpatient days of care in the VA. Following the onset of COVID-19 pandemic, monthly total acute care antibiotic use increased from 533 DOT/1000 DP in 1/2020 to a peak of 583 DOT/1000 DP in 4/2020; during that month COVID-19 patients accounted for 13% of all DP (Figure). In subsequent months, total antibiotic use declined such that for the full year the change of antibiotic use from 2019 to 2020 (a decrease of 18 DOT/1000 DP) was similar to the rate of decline from 2015 to 2019 (mean decrease of 13 DOT/1000 DP; Table). The decreased DOT/1000 DP from 5/2020 to 2/2021 occurred even as the percentage of all DP due to COVID-19 peaked at 14 - 24% from 11/2020 to 2/2021.

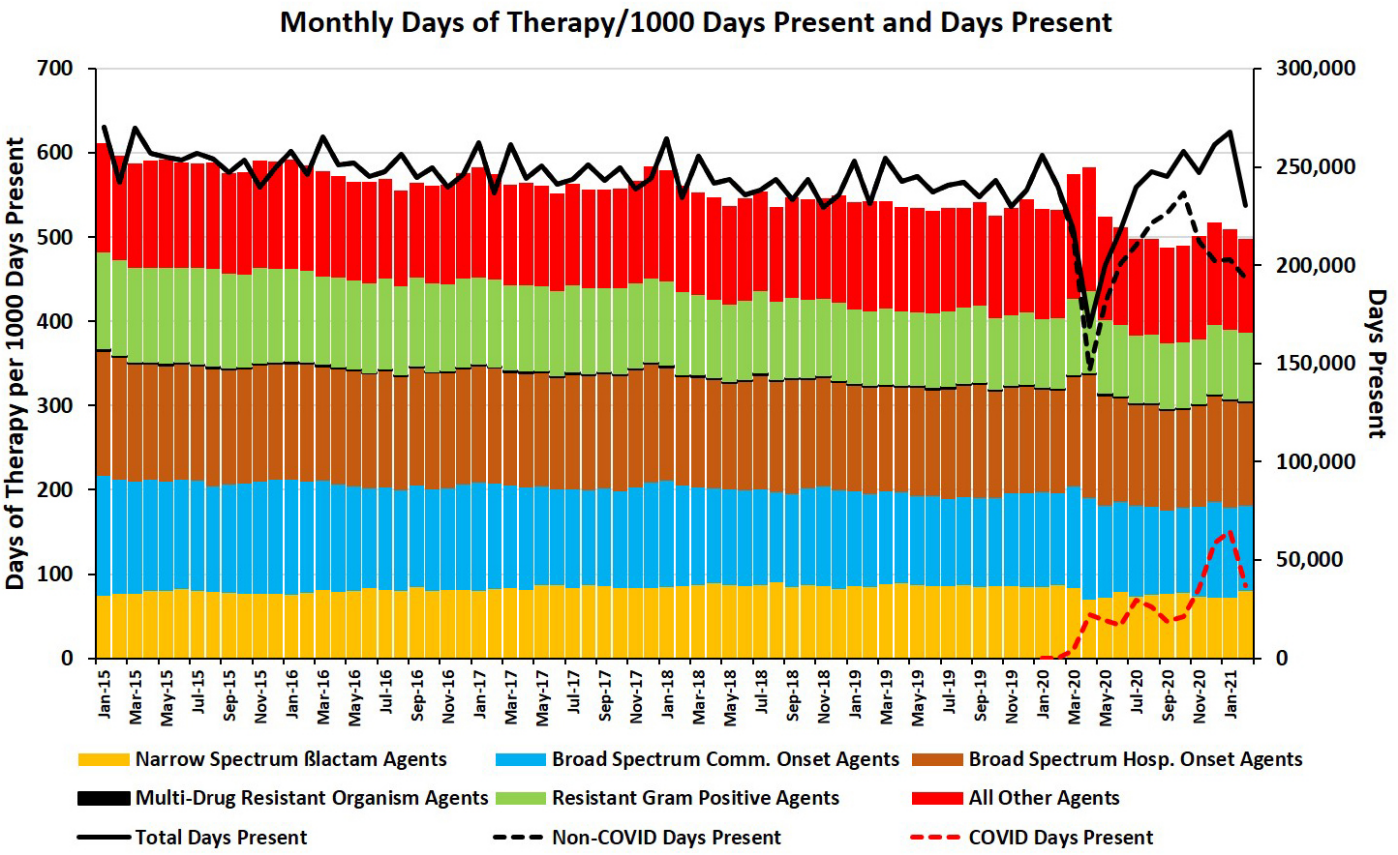

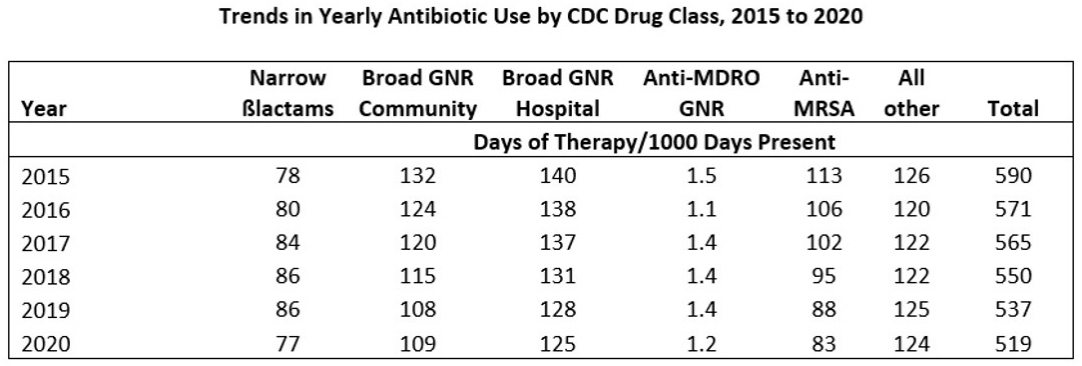

**Conclusion:**

Although rates of antibiotic use increased within the VA during the early phases of the COVID-19 pandemic, rates subsequently decreased to below previous baseline levels even as the proportion of COVID-19 DP spiked between 11/2020 and 02/2021. Although the degree to which the initial increase in antibiotic use is attributable to concerns of bacterial superinfection versus changes in case-mix (e.g., decreased elective admission) remains to be assessed, these data support the continued effectiveness of antimicrobial stewardship programs in the VA.

**Disclosures:**

**Matthew B. Goetz, MD**, Nothing to disclose

